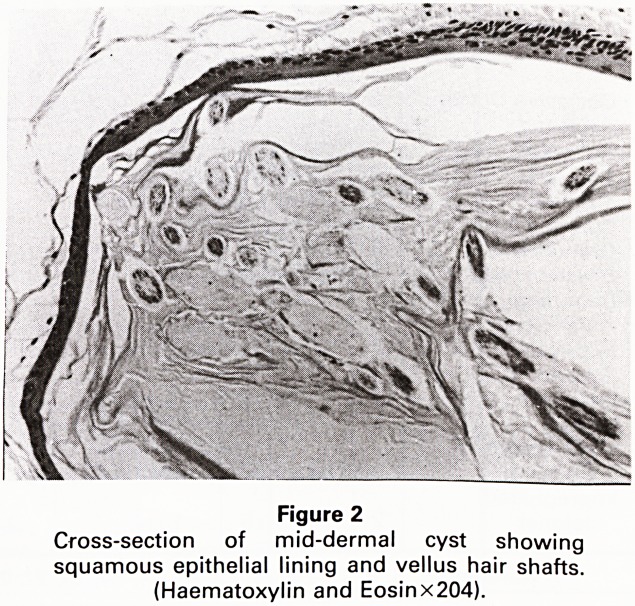# Eruptive Vellus Hair Cysts

**Published:** 1986-02

**Authors:** C. E. H. Grattan, R. R. M. Harman

**Affiliations:** Department of Dermatology, Bristol Royal Infirmary, Bristol, BS2 8HW; Department of Dermatology, Bristol Royal Infirmary, Bristol, BS2 8HW

## Abstract

A case of sporadic eruptive vellus hair cysts occurring in a young Chinese woman is described. Many of the lesions had an unusual and distinctive dark blue discolouration. Resolution, by spontaneous discharge of the encysted vellus hairs to the surface, was observed.


### Eruptive vellus hair cysts

**C. E. H. Grattan MB, BChir, MRCP. and R. R. M. Harman MB, BS, FRCP.**
Department of Dermatology, Bristol Royal Infirmary, Bristol, BS2 8HW.

### SUMMARY

A case of sporadic eruptive vellus hair cysts occurring in a young Chinese woman is described. Many of the lesions had an unusual and distinctive dark blue discolouration. Resolution, by spontaneous discharge of the encysted vellus hairs to the surface, was observed.

### INTRODUCTION

Eruptive vellus hair cysts were first reported by Esterly, Fretzin and Pinkus in 1977. They described two children who developed asymptomatic papular eruptions over the chest and mentioned two other cases, also children, who presented after submission of the original manuscript. Histology revealed a mid-dermal cyst lined by squamous epithelium containing keratinous material and vellus hairs. Lee, Kim and Kang (1984) have recently reported 11 cases from Korea and have reviewed the literature.

Typically multiple flesh-coloured, red or dark brown papules erupt on presternal skin, although other sites may be affected. The condition occurs in both sexes but appears to be confined to children and young adults. An autosomal dominant pattern of inheritance has been recorded in two kindreds (Piepkorn, Clark and Lombardi, 1981; Stiefler and Bergfeld, 1980). Burns and Calnan (1981) described a case in a Negro but there have been no reports of this condition in the Chinese.

**Figure 1**
Multiple papules on presternal skin.

### CASE REPORT

A 20 year old Chinese woman presented with multiple asymptomatic papules, measuring 1 to 3 mm in diameter, concentrated on the presternal and inframammary skin (Figure 1). Scattered lesions were also present on her abdomen and lumbosacral area. She had noticed them several years earlier but only sought medical advice when they increased in number and depth of pigmentation. No other member of her family was affected. The papules varied from the normal skin colour to deep blue and were palpable as tiny hard intradermal papules with some similarity to epidermal cysts or closed comedones. Histology of a lesion from the lumbosacral area showed a typical mid-dermal cyst lined with squamous epithelium containing vellus hair shafts (Figure 2). Hair bulb remnants and appendigeal structures were not identified on serial sections. Spontaneous improvement occurred over several months. Tufts of interwoven fine pigmented hairs were observed by the patient to extrude on to the skin surface when the cysts ruptured. The hairs were easily extracted and did not recur.

**Figure 2**
Cross-section of mid-dermal cyst showing squamous epithelial lining and vellus hair shafts. (Haematoxylin and Eosin×204).

### DISCUSSION

Bovenmyer (1979) observed communication of a cyst with the skin surface and postulated that the contents had been expelled by a process similar to transepithelial elimination, which occurs in pathological states such as elastosis perforans serpiginosa, perforating folliculitis and reactive perforating collagenosis (Mehregan, 1970). Mihara (1984) demonstrated that elastic fibres are eliminated through regenerated epidermis of human skin using histochemical, immunofluorescent and electron microscopic techniques. No abnormality was detected in the elastic fibres but it appeared that lymphocytes and histiocytes were playing an important role in their recognition and removal. As Bovenmyer has shown that vellus hairs may penetrate a cyst wall it is possible that the presence of this foreign material in the dermis triggers rejection of the cyst by a similar process. Unfortunately we were not able to examine an extruding cyst histologically as our patient declined further biopsies. However, there is little doubt that the cyst contents were being discharged from her clear description of the hair tufts which appeared as the lesions resolved.

The striking blue pigmentation may have been due to the melanin content of the encysted vellus hairs in the mid and deep dermis. It is likely that this physical sign would not be seen in a fair-haired Caucasian or a Negro skin as it would require both a darkly pigmented hair and a light skin to be apparent.

Although the condition has been described only once before in the British medical literature (Burns et al., 1981) it may be more common in young people than has been recognised. The asymptomatic and benign nature of the condition, together with the tendency towards spontaneous resolution, no doubt deter all but the most determined from seeking a specialist opinion and the dearth of textbook references hinder the clinician from making the correct diagnosis.

**ACKNOWLEDGEMENT**

We thank J W B Bradfield, Consultant Histopathologist, Bristol Royal Infirmary, for preparation of the histology.

**REFERENCES**

BOVENMYER, D. A. (1979) Eruptive vellus hair cysts. *Archives of Dermatology,* **115**, 338–339.

BURNS, D. A. and CALNAN, C. D. (1981) Eruptive vellus hair cysts. *Clinical and Experimental Dermatology,* **6**, 209–213.

ESTERLY, N. B., FRETZIN, D. F. and PINKUS, H. (1977) Eruptive vellus hair cysts. *Archives of Dermatology,* **113**, 500–503.

LEE, S., KIM, J-G. and KANG, J. S. (1984) Eruptive vellus hair cysts. *Archives of Dermatology,* **120**, 1191–1195.

MEHREGAN, A. H. Transepithelial elimination. Current Problems in Dermatology, Basel, Switzerland, S. Karger, 1970, **3**, 124–147.

MIHARA, M. (1984) Transepithelial elimination of elastic fibres in the regenerated human epidermis. *British Journal of Dermatology,* **110**, No. 5, 547–554.

PIEPKORN, M. W., CLARK, L. and LOMBARDI, D. L. (1981) A kindred with congenital vellus hair cysts. *Journal of the American Academy of Dermatology,* **5**, 661–665.

STIEFLER, R. E. and BERGFELD, W. F. (1980) Eruptive vellus hair cysts—an inherited disorder. *Journal of the American Academy of Dermatology,* **3**, 425–429.

## Figures and Tables

**Figure 1 f1:**
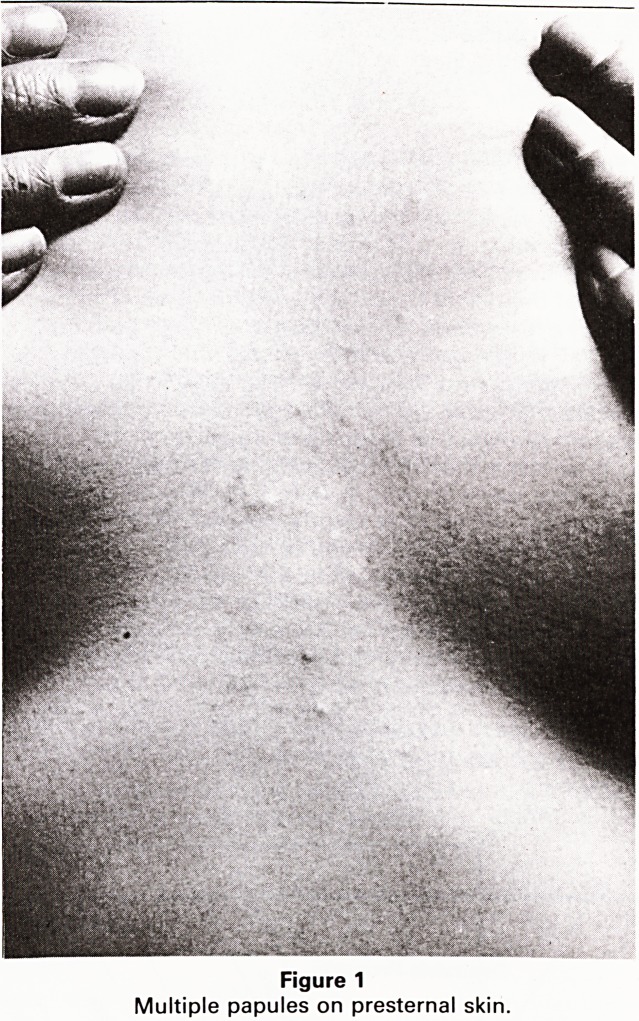


**Figure 2 f2:**